# Semi-Automated Analysis of Digital Photographs for Monitoring East Antarctic Vegetation

**DOI:** 10.3389/fpls.2020.00766

**Published:** 2020-06-09

**Authors:** Diana H. King, Jane Wasley, Michael B. Ashcroft, Ellen Ryan-Colton, Arko Lucieer, Laurie A. Chisholm, Sharon A. Robinson

**Affiliations:** ^1^Centre for Sustainable Ecosystem Solutions, School of Earth, Atmospheric and Life Sciences, University of Wollongong, Wollongong, NSW, Australia; ^2^Global Challenges Program, University of Wollongong, Wollongong, NSW, Australia; ^3^Antarctic Conservation and Management, Australian Antarctic Division, Kingston, TAS, Australia; ^4^Research Institute for the Environment and Livelihoods, Charles Darwin University, Alice Springs, NT, Australia; ^5^School of Technology, Environments and Design, College of Sciences and Engineering, University of Tasmania, Hobart, TAS, Australia

**Keywords:** Antarctica, moss, vegetation cover, vegetation health, climate change, monitoring, OBIA, RGB

## Abstract

Climate change is affecting Antarctica and minimally destructive long-term monitoring of its unique ecosystems is vital to detect biodiversity trends, and to understand how change is affecting these communities. The use of automated or semi-automated methods is especially valuable in harsh polar environments, as access is limited and conditions extreme. We assessed moss health and cover at six time points between 2003 and 2014 at two East Antarctic sites. Semi-automatic object-based image analysis (OBIA) was used to classify digital photographs using a set of rules based on digital red, green, blue (RGB) and hue-saturation-intensity (HSI) value thresholds, assigning vegetation to categories of healthy, stressed or moribund moss and lichens. Comparison with traditional visual estimates showed that estimates of percent cover using semi-automated OBIA classification fell within the range of variation determined by visual methods. Overall moss health, as assessed using the mean percentages of healthy, stressed and moribund mosses within quadrats, changed over the 11 years at both sites. A marked increase in stress and decline in health was observed across both sites in 2008, followed by recovery to baseline levels of health by 2014 at one site, but with significantly more stressed or moribund moss remaining within the two communities at the other site. Our results confirm that vegetation cover can be reliably estimated using semi-automated OBIA, providing similar accuracy to visual estimation by experts. The resulting vegetation cover estimates provide a sensitive measure to assess change in vegetation health over time and have informed a conceptual framework for the changing condition of Antarctic mosses. In demonstrating that this method can be used to monitor ground cover vegetation at small scales, we suggest it may also be suitable for other extreme environments where repeat monitoring via images is required.

## Introduction

Climate change has caused range shifts in vegetation communities toward the poles and alpine regions, as well as changes in phenology and growth worldwide (IPCC, 2014; Pecl et al., 2017). In order to inform management of ecosystems, determine community dynamics and detect change in vegetation communities over time, vegetation must be monitored over the long-term (Howard-Williams et al., 2006; Barrett et al., 2008; Michaels and Power, 2011). Where fieldwork is difficult, dangerous, remote or expensive, it is much more challenging to ensure adequate monitoring. One such example is long-term monitoring of vegetation in Antarctica, where there are logistical difficulties relating to the expense of sending researchers to Antarctica, frequency and duration of visits, weather-related delays, field surveys conducted in the extreme cold and mechanical difficulties with equipment. In addition, protective regulations may limit certain methodologies e.g., the Antarctic Treaty System principles necessitate minimal destructive sampling of vegetation.

Plant productivity and growth is limited in the harsh Antarctic environment, due to the related subzero temperatures and lack of free water (Johansson and Thor, 2008; Wasley et al., 2012). Plant life on the Antarctic continent is dominated by lichens and mosses and restricted to ice-free regions, which comprise less than one percent of the continent (Lee et al., 2017; Robinson et al., 2018). Given that Antarctic moss turfs (see Supplementary Figure 1 for examples) span areas of centimeters to meters and are comprised of mosses of small size (<15 cm depth) and high shoot density (550–900 shoots per cm^2^) (Wasley et al., 2006), some vegetation monitoring techniques typically applied at larger scales for remote sites may not be suitable. For example, remote sensing techniques can repeatedly monitor the state of Antarctic vegetation at distinct time points, which reduces time spent in the field and minimizes destructive impacts upon the vegetation communities, in accordance with Antarctic Treaty System principles. However, due to the small size of the moss turfs, even the highest resolution satellite imagery available (2.2 m pixel resolution WorldView-2) is unsuitable for vegetation studies in these areas (Malenovský et al., 2017). Digital photography with very high (1–4 m) and ultra-high (<1 m) spatial resolution, taken from the air (via unmanned aircraft systems, aka UASs) is ideal (Turner et al., 2012, 2014, 2018; Bollard-Breen et al., 2015; Malenovský et al., 2017), however, it is expensive to obtain and operate suitable UASs in Antarctica, they require a licensed pilot (and thus increased logistics support and funding), and weather conditions make their use difficult in certain locations. The small size of the mosses, in addition to the often prohibitive costs of obtaining suitably scaled aerial/UAS photography in Antarctica, means that digital photography using handheld cameras may still be the most suitable low-cost option for long-term monitoring of many Antarctic vegetation communities. Handheld digital photography is very easy to accomplish, and can be done by anyone with a camera and GPS locations of quadrats, making repeat monitoring much more feasible. The challenge lies in determining the balance between monitoring parameters and aligning these with current sensor technology, whilst future-proofing methods to enable the spatio-temporal monitoring necessary to assess the impacts of global climate change.

Standard red, green, blue (RGB) digital photography has been successfully utilized in vegetation studies to determine vegetation cover (Bennett et al., 2000; McCarthy and Zaniewski, 2001; Booth et al., 2005a; Laliberte et al., 2007b; Greenwood and Weisberg, 2009; Ko et al., 2009; Haywood and Stone, 2011; Kim et al., 2011), vegetation type (Ehlers et al., 2006; Lathrop et al., 2006; Luscier et al., 2006; Yu et al., 2006; Hájek, 2008; Greenwood and Weisberg, 2009; Laliberte et al., 2010; Michel et al., 2010; Cserhalmi et al., 2011; Whiteside et al., 2011) and vegetation changes over time (Bennett et al., 2000; Ehlers et al., 2006; Cserhalmi et al., 2011). As vegetation communities have complex characteristics, with patches varying in size, internal homogeneity and discreteness, it makes sense to analyze these communities based on combinations of their spatial and spectral patterns (Blaschke and Strobl, 2001). Object-based image analysis (OBIA) is a useful technique to analyze such communities, with images being segmented into relatively homogeneous areas to create meaningful objects for analysis (Blaschke and Strobl, 2001; Liu and Xia, 2010), with rules developed to isolate elements of interest. These rules are objective, and not prone to the errors associated with subjective human perception and interpretation of vegetation patterns (Elzinga et al., 1998; Van Coillie et al., 2014), making them ideal for use for long-term monitoring applications.

Numerous studies have used OBIA methodology for the evaluation of vegetation community characteristics in airborne and space-borne imagery, with advances in analytical software and technology leading to a marked increase in the usage of effective image segmentation methods (Hay et al., 2005; Bunting and Lucas, 2006; Conchedda et al., 2007; Laliberte et al., 2007a; Jobin et al., 2008; Pringle et al., 2009; Berberoglu et al., 2010; Blaschke, 2010; Laliberte and Rango, 2011; Torres-Sánchez et al., 2015). However, few studies have used OBIA for percent cover analysis of vegetation in near-surface field photographs (Luscier et al., 2006; Laliberte et al., 2007b; Zhang et al., 2007; Michel et al., 2010), with only one study using OBIA to measure vegetation percent cover in a polar region (Chen et al., 2010). Classification methods using fuzzy logic based on neighboring pixel values have been utilized in vegetation classification analyses, leading to high classification accuracy (Luscier et al., 2006; Laliberte et al., 2007b), however, these methods can lead to confusion between vegetation classes (Chen et al., 2010). Rule-based classification of vegetation in an Arctic study was successfully performed with high accuracy, using both RGB bands and HSI (hue, saturation, and intensity) bands (transformed from the original RGB images) threshold values (Chen et al., 2010), as HSI transformations can be very effective for vegetation classifications (Laliberte et al., 2007b).

For sensitive and remote sites, and particularly for short stature vegetation, the use of OBIA to assess digital RGB photographs of quadrats enables vegetation cover analyses to be performed in a timely and non-destructive manner, saving on field costs and labor. Digital photographs can also be easily stored and provide an invaluable source of data for other monitoring applications. Here we introduce an object-based image analysis methodology to assess short stature vegetation percent cover and compare its results to those of two other methods: manual digitization and expert visual estimates. We then assess temporal change using Antarctic moss turfs as a model short stature plant community.

## Materials and Methods

A long-term vegetation monitoring system was established in 2003 at two sites in the Windmill Islands, East Antarctica: Antarctic Specially Protected Area (ASPA) 135 (site A2) and Robinson Ridge (site RR) (as per Wasley et al., 2012; Malenovský et al., 2017; Robinson et al., 2018) (see Supplementary Figure 1 for site maps). These sites exhibit some of the most extensive moss beds in the region, and have been extensively described in Lovelock and Robinson (2002), Dunn and Robinson (2006), Wasley et al. (2012), and Turner et al. (2014). Sixty permanent 25 × 25 cm quadrat locations have been repeatedly monitored in three vegetation communities at these sites (10 quadrats each in Bryophyte, Transitional and Lichen communities at each site) between 2003 and 2014 (Robinson et al., 2018). These communities occur along a moisture gradient, the wettest dominated by bryophytes (Bryophyte community, approximately 90% live bryophyte cover); the driest dominated by lichens overgrowing moribund moss (Lichen community, <5% live bryophyte cover); and the community in between comprised of both types of vegetation (Transitional community, approximately 40% live bryophyte cover) (Ryan-Colton, 2007). Digital RGB photographs of each quadrat were taken in Jan–Feb five times between 2003 and 2013, as well as the Bryophyte community quadrats in 2014 (Supplementary Table 1). Unfortunately, the Lichen community could not be analyzed digitally for vegetation cover, as macrolichens, which dominated cover in this community, were not suitable for this analysis method. This was due to difficulties differentiating between vegetation cover types in this community using only RGB spectral characteristics, as the range of different black, gray and white lichens are visually very similar to black, gray and white rocks.

As the moss beds are sensitive vegetation, easily damaged by trampling and disturbance, care had to be taken to prevent standing on the moss, thus handheld photographs had to be taken from above the quadrat whilst standing on the nearest rocks to the quadrat location. All care was taken to stand in the same position each subsequent year for photography, however, differences in camera height and position were inevitable between different quadrats and years. Different cameras were also used in different field seasons, as technology improved over time (Supplementary Table 1). All photography acquired over time kept settings in mind so to best replicate consistency across different cameras (composition, adjustments for exposure, etc.), but exact duplication of the camera setup is not necessary in order to detect change over time (Rogers et al., 1983; Hall, 2002). Reference photos from the baseline 2003 field season were used to correctly reposition the physical metal quadrat each subsequent field season, however, as frost heave causes moss to move within a quadrat over time, the physical positions of the quadrats do change slightly between years, thus direct pixel to pixel change detection over time is problematic. Determining the change in percent cover of moss within each quadrat was therefore the most efficient method to assess vegetation health over time.

### Image Analysis

**FIGURE 1:**
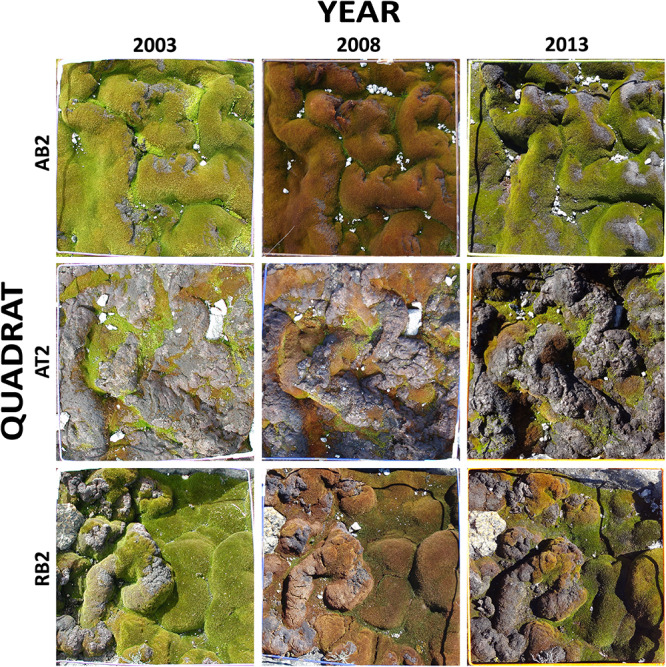
Test mosaic of nine quadrat images from sites in the Windmill Islands, East Antarctica. The mosaic comprises photos of three quadrats, taken at 5 year intervals (2003, 2008, and 2013). AB2: ASPA 135 Bryophyte community quadrat 2, AT2: ASPA 135 Transitional community quadrat 2, RB2: Robinson Ridge Bryophyte community quadrat 2.

To assess the feasibility of using object-based image analysis for estimation of percent cover of short-stature vegetation communities, first a test image mosaic was created from nine digital quadrat photographs (Figure 1). Photographs taken at 5-year intervals (2003, 2008, and 2013) were included for each of three representative quadrats included in the test mosaic, out of the total 40 quadrats (excluding Lichen community quadrats) in the monitoring system (Figure 1). These images included moss in the full range of health states from bright green/olive green healthy moss, through to red/brown stressed moss and black/gray moribund moss. Moribund moss is moss that appears dead (gray to black in color) and has no evidence of chlorophyll presence in the leaves when examined microscopically (see inset images in Figure 8). Although appearing dead, moribund moss can sometimes, under ideal conditions, regenerate providing some healthy cells are present (Robinson et al., 2020).

The quadrat images were georeferenced using ArcGIS v 9.3 (ESRI, Redlands, CA, United States). The spatial resolution of the quadrat images changed with the varied positioning of quadrats within the images and improved camera quality over time, from 2048 × 1536 pixels in 2003 to 6000 × 4000 pixels in 2014 (Supplementary Table 1). All images were rectified to a pixel size of 0.0005 m, using nearest neighbor resampling to retain original pixel values for vegetation classification (Verbyla, 2002), allowing direct comparison of results between quadrats and across years.

As the area within each quadrat was the focus for vegetation classification, the physical quadrat itself, as well as the area outside the quadrat in each image, was masked out (“Quadrat polygons,” Supplementary Figure 2). As these images were simple RGB digital photographs, it was difficult to distinguish between some of the ground cover classes, which could typically be differentiated by spectral characteristics, for example: gray rock vs. gray moribund moss, black rock vs. black moribund moss vs. black macrolichens and white rock vs. white macrolichens. As health status of moss was the principal focus of monitoring, manual digitization was undertaken to mask out all ground cover classes in the quadrats except moss turf (e.g., rock, crustose and macro-lichens, snow and water) (“Rocks polygons,” “Crustose polygons,” “Macrolichen polygons,” “Snow polygons,” and “Water polygons,” respectively, Supplementary Figure 2). Some quadrats contained areas of very wet moss, which was so dark in color that it tended to be incorrectly classified as moribund moss, so these areas were also manually digitized and classified (“WetMoss polygons,” Supplementary Figure 2).

Many different methods exist for object-based image analysis, however, *eCognition* (Trimble, München, Germany) is the most common commercial software package capable of performing a multiscale image segmentation approach, ideal for land cover analysis (Blaschke, 2004). Therefore, to assess which parameters and rules would be most successful in classifying vegetation into categories of healthy, stressed or moribund, the image mosaic for the quadrats, as well as the associated masks, were imported into the eCognition OBIA software for analysis (Supplementary Figure 2).

#### Segmentation

Optimal settings for image object segmentation were determined using the Estimation of Scale Parameter (ESP) tool (Drǎguţ et al., 2010). This tool allows the user to improve object segmentation parameterization in eCognition by showing changes in local variance (LV, the mean of all standard deviations of a moving 3 × 3 pixel area across the image), and rate of change (ROC) across a range of scale parameters (Drǎguţ et al., 2010). Peaks in the ROC-LV graph indicate the most appropriate object levels for segmentation, and segmentation at each of these optimal levels can be visually assessed to determine which scale level is necessary to segment meaningful objects of interest for subsequent image classification. Multiresolution image segmentation was performed at different optimal scales, and the segmentation results were visually compared. Once optimal segmentation parameters were determined using the ESP tool, multi-resolution image segmentation was performed on the image mosaic, with scale parameter 27, shape 0.1 and compactness 0.5, to create image objects for classification. The manually digitized polygons created during pre-processing were included as thematic layers during image segmentation, in order to be used as masks for ground cover classification (Supplementary Figure 2).

#### Classification

Various object features were visually assessed to determine those best suited to delineating the three different health states of the moss turf (healthy, stressed, and moribund). Upon evaluation, the OBIA features found to be most useful for object classification included: pixel-based ratios of red, green and blue (RGB); Intensity and Saturation from the hue, saturation and intensity (HSI) color space (converted from RGB within the eCognition software); relative border to another class; and area of objects. Red, green and blue pixel ratios (to overall brightness) were used instead of mean R, G and B values, as ratios were less affected by different lighting conditions and different cameras (Supplementary Table 1) (Jensen, 2013).

To aid the visual assessment of thresholds between classes, object features were separated into 15 Jenks Natural Breaks categories, classes being determined by natural breaks between clusters in the data, maximizing the differences between classes and grouping similar values. The threshold was assessed visually and the value was determined as the digital value separating the most appropriate Jenks Natural Breaks categories. This value was then used as the threshold value for object classification within the eCognition ruleset.

The different red, green and blue pixel ratios alone were not enough to definitively separate the ground cover classes, however, when combined into Death index1 (Eq. 1) they were useful for classifying the majority of moribund moss, with high values indicating moribund moss. Death index2 (Eq. 2) was useful for distinguishing the remaining moribund moss, with lower values indicating moribund moss. A Stress index (Eq. 3) indicated stressed moss with high values indicating most stress, i.e., a higher contribution of red to overall object brightness. Conversely, a low Stress index combined with high pixel ratios of green indicated healthy moss. Both HSI Intensity and Luminance (Eq. 4, where PbR denotes “Pixel based Ratio of,” and R, G, and B are the red, green and blue bands, respectively), were found to be useful in distinguishing shadows (black, low Intensity and low Luminance) and small wind-blown rocks (white, high Intensity and high Luminance) (Trussell and Vrhel, 2008).

(1) $$D\,e\,a\,t\,h\,\,i\,n\,d\,e\,x\,1=\frac{\mathrm{PbR}\,B}{\left(\mathrm{PbR}\,G*\mathrm{PbR}\,R\right)}$$

(2) $$D\,e\,a\,t\,h\,\text{⁢}\,i\,n\,d\,e\,x\,2=\frac{\mathrm{HSI}\,\mathrm{Intensity}}{\left(\mathrm{HSI}\,\mathrm{Saturation}*\mathrm{PbR}\,R*\mathrm{PbR}\,G\right)}$$

(3) $$S\,t\,r\,e\,s\,s\,\,i\,n\,d\,e\,x=\frac{\mathrm{PbR}\,R}{\mathrm{PbR}\,G}$$

L⁢u⁢m⁢i⁢n⁢a⁢n⁢c⁢e=(0.2126*P⁢b⁢R⁢⁢R)+(0.7152*P⁢b⁢R⁢⁢G)+(0.0722*P⁢b⁢R⁢⁢B)

The classification process involved a series of manually refined rules determining which class an object should be assigned to, based on digital value thresholds, followed by further refinement of certain classes based on position and size of an object (Supplementary Figure 2). For example, if an object was surrounded by healthy moss, and the object was very small, it was likely to be a small wind-blown rock. Similarly, areas of shadow were reassessed depending on whether the border of an object was predominantly shared with moss of a particular health state, for example if an object was classified as shadow and shared more than 50% of its border with objects classified as healthy moss, it is likely that that area of shadow was actually healthy moss.

The results of the classification were assessed visually for each quadrat. Post-processing involved manual correction of some small misclassification issues, for example areas of wet healthy moss (very dark green, too dark for the classification algorithms to detect as live moss), not digitized in the pre-processing stage, and misclassified as moribund, were corrected upon expert visual assessment. Object statistics were exported, in order to obtain a final pixel area of each class, for later conversion to a final percent cover. A tif image was also exported to show the classification results for each quadrat. Finally, a polygon shapefile was exported with a field for class name, for later visualization of the classification.

### Accuracy Assessment

This monitoring methodology is designed to replace field estimation techniques of percent cover of vegetation, and it is therefore imperative to ascertain the accuracy of the automated classification process, including both the thematic accuracy of the classification as well as how the classification results compared with the most commonly used current field technique, the vegetation cover accuracy.

#### Thematic Accuracy

The subset of nine quadrat images used for data exploration and assessment was subsequently used for accuracy assessment of the rule-based classification. The map document created for the original image pre-processing was further used for manual classification of the mosaic by manual digitization of the various vegetation classes. Two of the 9 quadrat images in the mosaic were also independently digitized by a further three experts, in order to achieve a majority consensus of the classification of the vegetation types in the quadrats that were most difficult to assess. This majority consensus was used in the final manual classification shapefile, in addition to the other seven manually classified quadrats.

Following semi-automated classification, the classification result was imported into the pre-processing map document and compared with the manual classification. Manual and semi-automated classification results were both spatially joined to a grid of points spaced 0.005 m apart across the entire mosaic (one point per pixel in the mosaic, a total of 21608 points) to compare vegetation cover classifications, and the resulting table was exported as a database file for further analysis.

The statistics program SPSS v. 21 (IBM, New York, NY, United States) was used to compare the manual and rule-based classification results database files, and calculate the Conditional Kappa coefficient as a measure for thematic accuracy, to test individual category agreement between two different classifications (Congalton and Green, 2009). The Kappa analysis is a standard component of every accuracy assessment of image analysis, and is a measure of how well the image classification matches with the reference (ground truth) data (Congalton and Green, 2009). Values greater than 0.80 represent strong agreement (more than 80% of the image classification is the same as the ground truth data), 0.4 to 0.8 represent moderate agreement, and less than 0.4 represent poor agreement (Congalton and Green, 2009).

#### Vegetation Cover Accuracy

To compare with traditional visual percent cover estimates, three Antarctic moss experts were given the same three images to analyze, one from each year of sampling for a single quadrat (bottom row of mosaic Figure 1, quadrat RB2 from 2003, 2008, and 2013). The quadrat was overlaid with a 5 × 5 cm grid, mimicking the grid used in field estimates of percent cover throughout the Antarctic monitoring program. The experts assessed the percent cover of the various classes for each square in the grid, and the results were tallied to calculate total percent cover for each quadrat.

### Vegetation Change Assessment

Semi-automatic classification of digital photography was used to assess the change in vegetation health of continental Antarctic vegetation monitoring quadrats between 2003 and 2014. A total of 210 quadrat images from the Bryophyte and Transitional communities at both sites were acquired over the six field seasons. Some quadrats in some years were snow-covered, and could not be used for vegetation classification (10 photographs out of 220 over 6 years). The Transitional community was not photographed in 2014 due to field time constraints.

Image analysis occurred as above, including georeferencing, preprocessing, and semi-automated object-based image analysis for classification of vegetation health. A small number of photographs had unforeseen classification issues, such as areas of wet moss which were not immediately apparent in the preprocessing stage. Following manual correction, the output was saved, and a note made that this photograph had been manually corrected. This process was required for 47 photographs out of the entire set of 210 photographs of quadrats classified from the six field seasons, particularly those from 2011 and 2014 due to snow and wet moss.

Vegetation cover change over time was analyzed using a non-parametric Friedman Test in IBM SPSS v25 (IBM Corp., Armonk, NY, United States) for each vegetation cover category. Pairwise comparisons were performed (IBM SPSS v25) with a Bonferroni correction for multiple comparisons.

## Results

### Image Analysis

#### Segmentation

**FIGURE 2:**
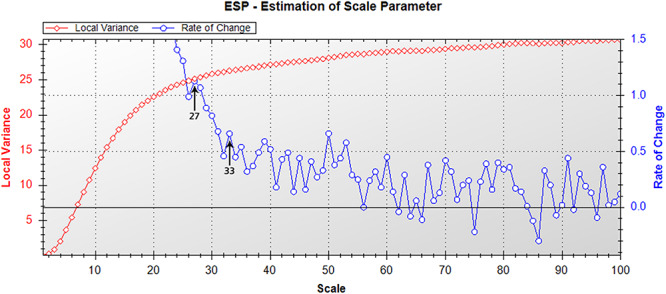
Estimation of scale parameter tool output (Drǎguţ et al., 2010) for the nine quadrat image mosaic. Graph depicts changes in local variance (LV, red) and rate of change (ROC, blue) with increasing scale parameter for segmentation of the moss quadrat test mosaic. Peaks in the ROC-LV graph indicate the most appropriate object levels for segmentation. Suitable scales for image segmentation for the mosaic were 27 or 33, as indicated by arrows.

**FIGURE 3:**
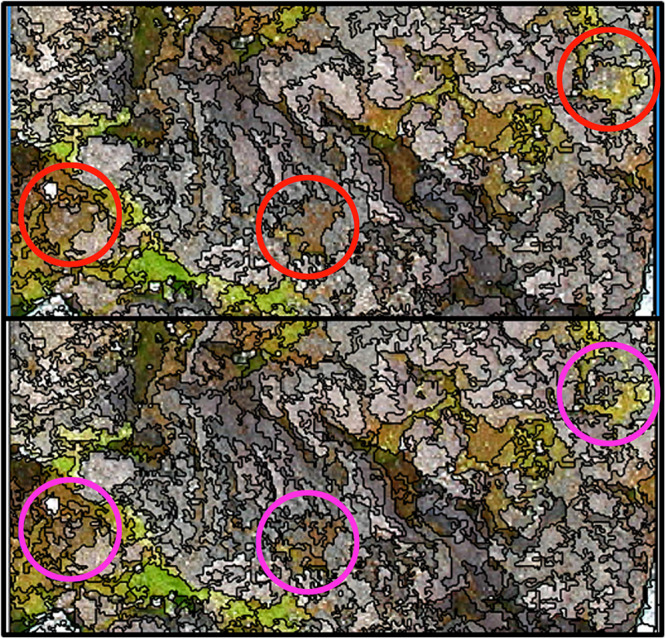
Comparison of scale parameters 33 (top) and 27 (bottom). Scale 27 was chosen as optimal for image segmentation, as it separated out the smaller areas of healthy, stressed and moribund moss, which formed mixed objects when the image was segmented with scale 33, as seen in the three circled areas.

The Estimation of Scale Parameter (ESP) tool determined optimal segmentation scales for the test mosaic of 27 and 33 for peaks in the rate of change (Figure 2). These values were therefore identified as optimal scale parameter settings for segmentation of the mosaic, with a scale of 27 ultimately selected as optimal for image segmentation, as there were more objects containing mixed vegetation categories when segmented with a scale of 33, which were separate objects when segmented with a scale of 27 (Figure 3). Scales higher than 33 were found to create objects that were too large for appropriate separation of vegetation types.

#### Classification

Classification of the image mosaic was an iterative process, with rules using thresholds to classify objects into intermediary classes, reclassifying objects, merging objects, looping classifications and final classification into final classes (Supplementary Figure 3).

**FIGURE 4:**
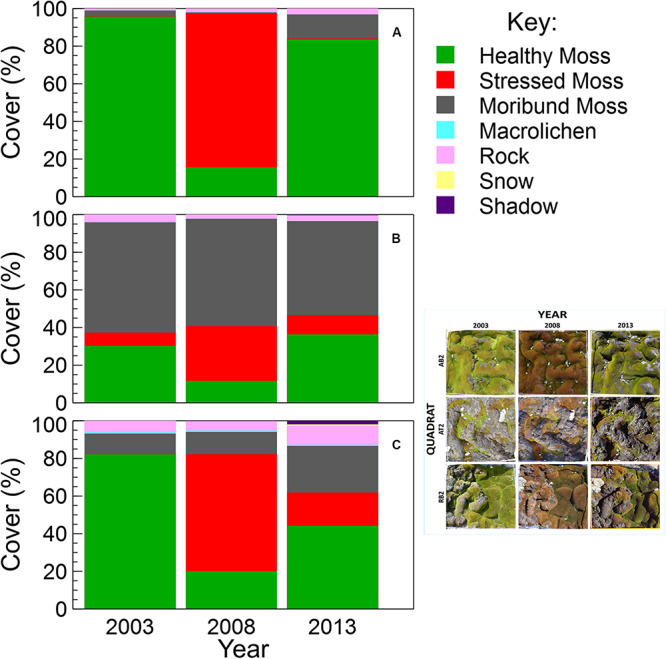
Comparison of vegetation percent cover within each quadrat in the test mosaic (Figure 1; inset at right): **(A)** AB2, **(B)** AT2, and **(C)** RB2, at 5-year intervals from 2003 to 2013, assessed using semi-automated object based image analysis.

Using image segmentation and classification of the test mosaic of nine quadrat images, the total estimated percent cover of each category across the entire mosaic was: 45.3% healthy moss, 23.7% stressed moss, 26.5% moribund moss, 4.0% rock, 0.2% lichens, 0.1% snow and 0.3% shadow. There were no water, wet moss or unclassified pixels in the test mosaic. Percent cover was also calculated for each of the nine quadrats (Figure 4).

### Accuracy Assessment

#### Thematic Accuracy

When comparing the semi-automated classification results with the manually digitized classification, the total classification accuracy was 84%, with an overall Kappa value of 0.76 (Table 1). The Conditional Kappa values ranged from 0.67 for stressed moss to 0.95 for rock. The greatest errors of omission (i.e., areas incorrectly omitted from a class) were 25% moribund moss and 14% stressed moss. The greatest errors of commission (i.e., areas incorrectly classified in a class) were 26% stressed moss and 17% healthy moss.

**TABLE 1:** Comparison of test mosaic classification by eCognition and manual classification by expert.

**(a)**		**Manual classification**							
		**Healthy moss**	**Stressed moss**	**Moribund moss**	**Lichens**	**Rock**	**Snow**	**Shadow**	**Total**
eCognition Classification	Healthy moss	38.03	2.13	5.16	0.00	0.25	0.00	0.00	**45.57**
	Stressed moss	3.24	17.54	2.89	0.00	0.06	0.00	0.00	**23.74**
	Moribund moss	1.14	0.67	24.27	0.00	0.12	0.00	0.00	**26.20**
	Lichens	0.01	0.00	0.01	0.15	0.00	0.00	0.00	**0.17**
	Rock	0.12	0.03	0.06	0.00	3.78	0.00	0.00	**3.99**
	Snow	0.01	0.00	0.00	0.00	0.00	0.08	0.00	**0.09**
	Shadow	0.12	0.05	0.08	0.00	0.02	0.00	0.00	**0.26**

	**Total**	**42.66**	**20.42**	**32.46**	**0**.**16**	**4.21**	**0.08**	**0**	**100**

**TABLE:** 

**(b)**	**Errors of omission**	**Errors of commission**	**Conditional kappa**
Healthy moss	11%	17%	0.71
Stressed moss	14%	26%	0.67
Moribund moss	25%	7%	0.89
Lichens	6%	11%	0.89
Rock	10%	5%	0.95
Snow	6%	11%	0.89
Shadow	–	100%	0.0

**Total accuracy**			**84%**

**Total kappa**			**0.76**

***(a)** Error matrix (%), **(b)** Accuracy assessment (%). Conditional Kappa calculated as per Congalton and Green (2009).*

#### Vegetation Cover Accuracy

**FIGURE 5:**
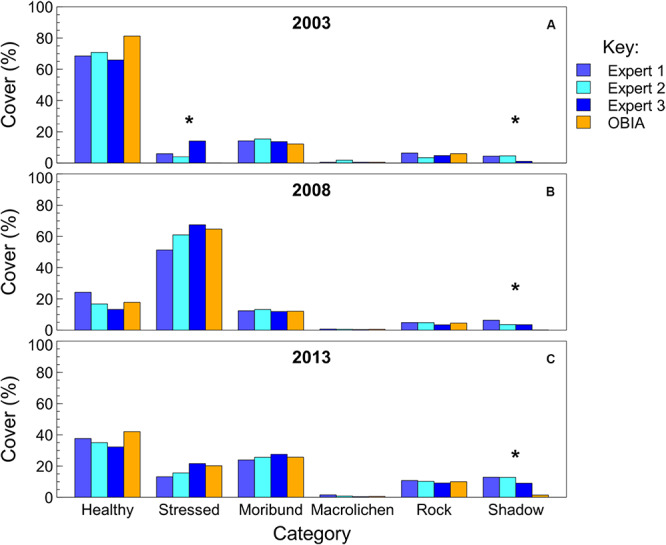
Comparison of percent cover estimates from three experts and the semi-automated object-based image analysis (OBIA). Quadrat images assessed were the bottom row of the mosaic in Figure 1, from **(A)** 2003, **(B)** 2008, and **(C)** 2013. Asterisks indicate significant differences between observers within each class in each year.

When comparing the semi-automated classification with that of three experts using traditional percent cover estimation techniques, the semi-automated classification results fell within the range of expert percent cover estimates for the majority of classes across the three quadrats (Figure 5). The average ranges between expert observers across the three quadrats were 7% for healthy moss, 12% for stressed moss, 2% for moribund moss, 1% for macrolichen, 2% for rock, and 3% for shadow. The semi-automated classification results were not significantly different from the experts for healthy moss, moribund moss, rock or lichens, across all three quadrats. Stressed moss was not significantly different between experts and semi-automated classification when there was more than 15% present, however, in quadrat R2B2003 the percent cover estimates varied significantly [*X*^2^(_4_,*_N_*_= 24_) = 31.19, *p* ≤ 0.0001], with semi-automated classification estimating 0% stressed moss, and the experts ranging from 4 to 14%. Shadow was significantly underestimated in all quadrats in the semi-automated classification [R2B2003: *X*^2^(_4,_ *_N_*_= 10_) = 13.32, *p* = 0.01; R2B2008: *X*^2^(_4,_ *_N_*_= 13_) = 14.34, *p* = 0.006; R2B2013: *X*^2^(_4,_ *_N_*_= 36_) = 30.79, *p* ≤ 0.0001].

### Vegetation Change Assessment

**FIGURE 6:**
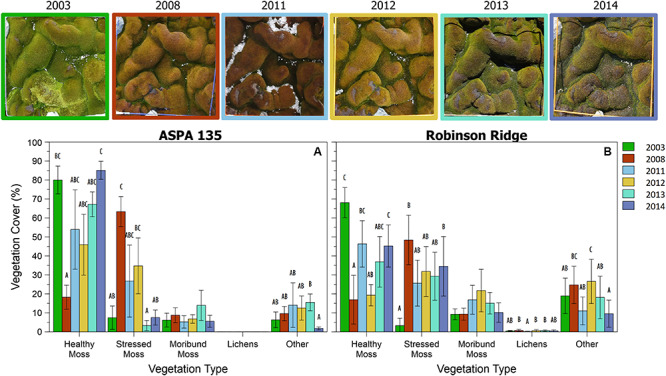
Trends in Bryophyte community vegetation cover from 2003 to 2014 at two sites in the Windmill Islands, A2 **(A)** and RR **(B)**. Photographs depict a representative quadrat (site A2, quadrat B3) at each of the six time points. Data are mean percent cover of vegetation ± 95% CI. Different letters denote significant differences between years per vegetation type.

**FIGURE 7:**
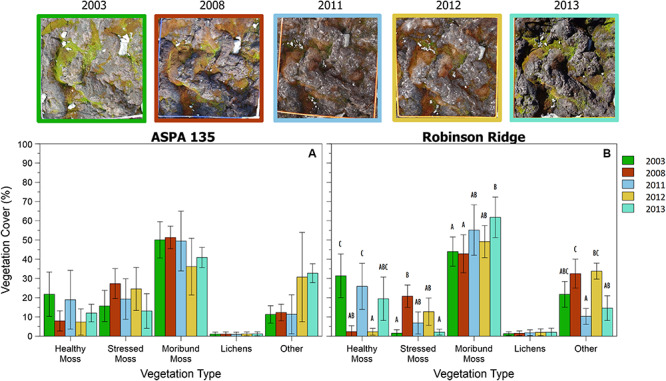
Trends in Transitional community vegetation cover from 2003 to 2013 at two sites in the Windmill Islands, A2 **(A)** and RR **(B)**. Photographs depict a representative quadrat (site A2, quadrat T2) at each of the five time points. NB: Transitional communities were not monitored in 2014. Data are mean percent cover of vegetation ± 95% CI. Different letters denote significant differences between years per vegetation type.

This method was successfully applied to all bryophyte (Robinson et al., 2018) and transitional community quadrats at six time points between 2003 and 2014 (Figures 6, 7). Vegetation health in both communities at ASPA 135 and Robinson Ridge experienced a general decline in 2008, followed by recovery by 2014 (Figures 6, 7). Healthy moss cover declined in 2008 to less than half of the 2003 cover at both sites in both communities (Figures 6, 7). This decline in healthy moss was associated with an equivalent significant increase in stressed moss cover from 2003 to 2008, in both communities at each site (Figures 6, 7). The amount of subsequent recovery differed between sites. ASPA 135 quadrats completely recovered to 2003 baseline levels of health by 2014 in the Bryophyte community (Figure 6), whilst no significant change over time was observed in the Transitional community (Figure 7). However, Robinson Ridge quadrats still appeared to be recovering in 2014, with a 10-fold increase (3% in 2003 to 35% in 2014) of stressed moss in the Bryophyte community (Figure 6), and an increase of moribund moss from 42% to 62% between 2003 and 2014, respectively, in the Transitional community (Figure 7). The cover of other abiotic factors, particularly snow, was variable between years (Figures 6, 7).

## Discussion

Our results suggest that semi-automated classification using object-based image analysis (OBIA) is a useful tool for quantifying percent cover estimates of vegetation in sites where fieldwork can be difficult, particularly for sensitive ground cover vegetation at the small scale where either consistent photographs have been taken over time or marked photopoints are available, so that new images can be obtained. The results confirm that semi-automated OBIA estimates of vegetation percent cover are within the range of visual estimation of cover by experts, and OBIA vegetation cover estimates can be used to objectively assess moss health changes over time.

Comparing the accuracy of methods, semi-automated classification and manual classification reference data had a thematic accuracy of 84% and a kappa value of 0.76. The thematic accuracy here is a conservative estimate, as thematic errors determined on a pixel-by-pixel basis may be present but still have little effect on overall percent cover estimates. For visual estimates, the experts, who were all familiar with the Windmill Islands vegetation and its various health states, all varied in their vegetation percent cover estimates. In general, between observer variability is known to be quite high for visual estimates of percent cover, regardless of the ecosystem being studied (Elzinga et al., 1998; Van Coillie et al., 2014). The semi-automated OBIA percent cover estimates fell within the range of expert estimates for the majority of classes across all three quadrats, with the only differences being significantly less shadow and significantly less stressed moss detected by the OBIA when it was present in less than 15% of a quadrat. Semi-automated classification significantly strengthens the analysis, as the differences in shadow estimates were due to the ability of the object-based image analysis software to use the red and green pixel ratios to assess whether a shadow was likely to contain healthy, stressed or moribund moss in areas of the image that were too dark for the human observers to judge in photographs. Some shadows remained too dark for determination of vegetation health within the shadow, with this typically occurring in frost-heave crevices. Even by eye in the field it is very difficult to identify with any level of accuracy the composition of vegetation within these crevices. Some issues with shadow in images may be reduced by further standardizing lighting conditions in the field (e.g., only take photos when cloudy), however, this is impractical to achieve in this remote environment, where field time is limited and opportunistic. The differences in stressed moss appeared to occur in areas where stressed moss mixed with healthy moss. In this case, there was sufficient stressed moss for human observers to determine a certain percentage stressed in a grid estimate, but not enough to override the predominance of healthy moss within an object in the object-based image analysis.

Standard field methods of percent cover estimation can overcome problems related to lighting conditions. In the field, it is possible to change viewing position to be able to see areas that may be obscured or difficult to see in a photo, and it is also possible to add extra light if needed in order to see into shadowy areas of a quadrat. However, the estimate of vegetation percent cover is subjective among human observers, and the accuracy of observers’ estimates is rarely estimated (Booth et al., 2005a; Gorrod and Keith, 2009). Field estimates of percent cover can be affected by adverse environmental conditions, such as the extreme cold in Antarctica, and the longer a researcher spends in the field, the less accurate observations may be, due to observer fatigue, the effects of changes in weather, time constraints and time of day (Bennett et al., 2000; Gorrod and Keith, 2009). Digital photography of quadrats required only one third of the field time required for traditional estimates of vegetation percent cover in the field. Due to the proposed length of this long-term monitoring study (25 years, with sampling originally planned at 5 year intervals), it is also likely that different researchers would perform the percent cover estimates each sampling period, thus each season would have a different observer bias. Thus, while our semi-automatic classification has only been assessed against expert manual classification of the same images and not field based estimates, it is likely that field based classification would be more variable and hence less reliable.

Based on the expert assessments in Figure 5, the estimation of healthy versus stressed moss cover appears to be the most subjective between observers. As this study involves classification using vegetation color to determine vegetation category (healthy = green, stressed = red and moribund = white/brown/black), it is important to note that color perception varies between observers, particularly between genders and different age groups, and this could affect vegetation percent cover assessments (Booth et al., 2005b; Abramov et al., 2012). Color perception is also highly dependent upon the variance and mean of surrounding colors (Brown and MacLeod, 1997), thus the assessment of whether or not a particular area of vegetation is healthy, stressed or moribund can also change for a single observer. It is difficult to maintain the same vegetation health categorization when the dominant color changes between quadrats, as well as being difficult to determine boundaries between categories when the vegetation varies along a health/color gradient. Using semi-automated classification based on RGB digital values helps to prevent these problems, as the classification involves a rule set using algorithms to make objective assessments of vegetation health, and the accuracy is sufficient to assess vegetation health changes over time.

To ensure long-term monitoring was able to include all of the digital photographs acquired in earlier seasons, it was imperative that the methodology developed be as robust as possible in order to be able to analyze digital RGB photographs from different years taken in different weather and acquisition conditions and at different resolutions. To achieve a conservative estimate of change over time, the rule set was designed to prevent overestimates of stressed and moribund moss, thus causing some errors with misclassification of stressed and moribund moss as healthy. Reference data introduces additional error and uncertainty in vegetation studies, thus is never perfect (Congalton and Green, 2009), and some error in the accuracy assessment was expected, due to the difficulty in defining the boundaries between vegetation health categories for such small and thin individual moss shoots. It can be difficult to define boundaries in nature and the vegetation health categories are usually found as a gradient between healthy and stressed, stressed and moribund or even healthy and moribund. The use of manual digitization as reference data is also only an approximation, due to the limitations of delineation using geographical information systems (GIS), so some geometric errors are inevitable and subject to edge effects (Bennett et al., 2000; Fensham and Fairfax, 2002; Aksoy et al., 2010). We attempted to increase the accuracy of the manual reference data by having multiple experts digitize the same portion (two quadrat images) of the mosaic to obtain a majority agreement, however, manual digitization is time consuming and due to time constraints it was not possible to have more than one researcher digitize the entire mosaic. The highest percent cover error in the error matrix was 5.16% of manually classified moribund moss being classified as healthy moss in the semi-automated classification, and this is likely due to areas of mixed vegetation health, where healthy moss shoots grow up through areas of moribund moss. It could also have been caused by the similarity in colors between some light-colored areas of moribund moss and certain areas of healthy moss which were moist enough to reflect sunlight, thus appearing light in color.

**FIGURE 8:**
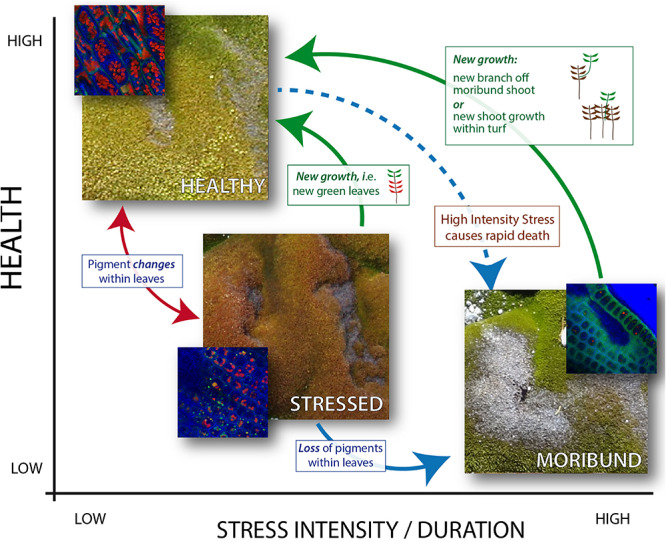
Diagrammatic representation of East Antarctic moss health state relationships. The intensity or duration of stress (*x* axis) impacts the health state (*y* axis). Solid arrows indicate observed heath state changes (2003–2014) of monitoring moss health in the Windmill Islands, with red and blue arrows indicating pigment changes within the cells of an individual plant, while green arrows indicate growth of new healthy leaves/plants at a turf level. Note that the three images show the same area of moss in different years, and that the moribund moss is the gray moss in the lower right image. Inset images (blue) depict confocal microscope images of cells at each stage of moss health, with bright red chloroplasts obvious in healthy moss but less clear in stressed moss and absent in moribund moss. Extreme high intensity stress is thought to cause immediate state change from healthy to moribund (dashed blue arrow), however, evidence for this is only anecdotal.

Once the methodology protocol was developed, it was applied to the full data set to assess vegetation health change over time (Figures 6, 7). Moss health state changes were observed between healthy, stressed and moribund over the 11 years of monitoring at ASPA 135 and Robinson Ridge (Figure 8). As described by Robinson et al. (2018) and shown here, both sites experienced a marked increase in stress and decline in health in 2008, in both the Bryophyte and Transitional communities. This was likely due to an unusual occurrence of freezing rain in that season (Robinson et al., 2018). The significant shift in moss color from green (healthy) in 2003 to red (stressed) in 2008 (Figures 6, 7) was followed by recovery of health by 2013–2014, although associated with increased stressed moss in the Robinson Ridge Bryophyte community (Figure 6B), and increased moribund moss in the Robinson Ridge Transitional community (Figure 7B). Our methodology using handheld digital photographs allows assessment of short and long term moss stress at a community level, prior to species-level changes in response to such stress (Robinson et al., 2018). We have seen resilience in these communities, as they have used changes in their protective pigments (becoming red in color) to respond to environmental stressors and subsequently “regreened” when conditions became more favorable. Despite this resilience, we have seen a remarkable increase in the submergence-intolerant species *Ceratodon purpureus* (Wasley et al., 2006, 2012; Robinson et al., 2018) and increased moribund moss.

Long-term monitoring coupled with image analysis improves our understanding of Antarctic vegetation health by underpinning a conceptual framework for how moss changes with stress and recovery (Figure 8). Antarctic moss health changes rapidly between stressed and healthy under ideal laboratory conditions, dependent on water availability, but further research is required to determine how long it takes for these changes under field conditions (Malenovský et al., 2015; Waterman et al., 2018). The color changes are caused by changes in the types of protective pigments (including anthocyanins and carotenoids) produced in the leaves of the moss (Post, 1990; Lovelock and Robinson, 2002; Waterman et al., 2018). ASPA mosses suffered stress resulting in pigment changes from green to red between 2003 and 2008, but this was reversed by subsequent loss of red pigments (red arrow) or growth of new leaves (green arrows). Both Robinson Ridge communities experienced the same stress response in 2008, however, the bryophyte community only partially recovered by 2014. In contrast, the transitional community demonstrates how mosses transition from green to red and finally moribund (red and blue arrows, Figure 8). These physiological, individual plant level changes occurred alongside species level changes, with an increase in the desiccation-tolerant mosses *Ceratodon purpureus* and *Bryum pseudotriquetrum* and a decline in the endemic *Schistidium antarctici* (Robinson et al., 2018). This appears to be linked to climatic changes such as regional drying and fewer days above zero degrees, in response to changes in the Southern Annular Mode (SAM) (Robinson et al., 2018). Further assessment of these changes through ongoing long-term monitoring will be vital for future management and conservation of these ecosystems (Bergstrom, 2017). There are, however, indications that moribund moss can regreen within 1 month, under the extreme flooding caused by recent Antarctic heatwaves (Robinson et al., 2020).

There are significant advantages of using semi-automated classification for long-term vegetation monitoring projects. The classification of vegetation health is objective and consistent, and each sampling season has the same rules, so all quadrats are assessed in the same way and valid comparisons can be made between photographs taken in different sampling periods over time, whether days, weeks or years apart. Digital photography of the vegetation quadrats means much less time needs to be spent in the field and all analyses can be completed in the comfort and safety of the laboratory (Bennett et al., 2000; Booth et al., 2005a). This is particularly useful in areas where fieldwork is difficult, dangerous or expensive. Digital photography of each quadrat can be accomplished in less than 5 min in the field, whereas field estimation of percent cover can take up to 15 min per quadrat. This reduces the time in the field for Antarctic vegetation monitoring from 15 to 5 h, which means that a single day per field site is feasible to complete all the required sampling each season. The digital photographs are easily stored, and are thus available for future retrospective studies examining new questions or using improved methods (Bennett et al., 2000; Booth et al., 2005a). For a long-term monitoring project, the rule development can take some time initially, as the rules used must be modified to suit that particular ecosystem. However, once a rule set is established with good accuracy for that ecosystem, this method is easily repeatable and consistent, and subsequent pre-processing and analysis of photographs is quite fast. Pre- and post-processing of the photographs does, however, require a trained expert in the field in order to accurately assess the vegetation types in the photographs, particularly when moss is wet. If quadrats are flooded with water or covered by snow, vegetation classification is difficult, whether using traditional field-based methods or photographs. Once the methodology protocol has been established, the combined field and lab work of OBIA can be as quick as, or quicker than, field estimations of percent cover, and is quicker than manual digitization, while reducing the effects of observer bias and allowing spatial and temporal comparisons of quadrats.

Recommendations to improve the accuracy include using a multispectral or hyperspectral camera that includes a near infrared band, enabling the use of the normalized difference vegetation index (NDVI), which would enable analysis of the Lichen community, as well as significantly reduce pre-processing time by allowing automated removal of non-vegetated areas of the image and better classification of vegetation health (Stow et al., 2004; Torres-Sánchez et al., 2015; Lawley et al., 2016). However, this would not allow comparisons with the earlier data collected in the monitoring study from 2003 to 2014. Accuracy could also be improved by standardizing the conditions for photography, for example ensuring that photos are not taken in direct sunlight, in order to reduce shadows and reflections within quadrat photographs, although time constraints with Antarctic fieldwork mean this is not always possible, and including color references in each image for color calibration (Kolyaie et al., 2019). The use of fuzzy classification methods in addition to rule-based methods may also increase the classification accuracy, although it would increase the time required for analysis as samples would have to be selected for each class in each image (Laliberte et al., 2007b). The use of multispectral very high resolution (VHR) satellite imagery has recently enabled the development of semi-automated satellite-based remote sensing methods of assessing Antarctic vegetation distribution (Jawak et al., 2015). However, those methods cannot yet distinguish the difference between vegetation types (such as moss, lichens, algae or cyanobacteria), and cannot determine vegetation distribution, both of which can be achieved using handheld digital photography. Using unmanned aerial systems (UAS’s) equipped with multispectral sensors is another good option for assessing vegetation distributions and health in the field, with minimal disturbance to the vegetation and the ability to monitor entire sites rather than just single quadrats, although ground observations are still required for data validation (Malenovský et al., 2017; Turner et al., 2018). It is likely that UASs will be increasingly utilized for vegetation monitoring in future, particularly in remote regions.

Here, we have shown that the use of object-based image analysis to classify digital photographs of quadrats provides an objective, repeatable, robust and fast method of assessing vegetation health over time. This method is particularly suited to remote, difficult to access locations with short stature vegetation communities, where field work and disturbance must be minimized, such as in polar, alpine and desert ecosystems. By analyzing long-term vegetation monitoring datasets with image analysis, we have informed a conceptual model for healthy, stressed and moribund moss in response to environmental stress and recovery. In East Antarctica it reveals that whilst moss health changes can be quite dynamic, a long-term decline in health is also apparent.

## Data Availability Statement

All data associated with this article is archived with the Australian Antarctic Data Centre (https://data.aad.gov.au/aadc/, doi: 10.26179/5eaf718e45748, doi: 10.26179/5eaf945e9b1ef, and doi: 10.4225/15/59fbbb909cf71).

## Author Contributions

JW and SR conceived the study. SR, JW, ER-C, and AL performed the field work. ER-C conducted the pilot study. DK designed the methods and analyzed the data. MA, AL, LC, and SR assisted with the method development. MA assisted with the data analysis. DK wrote the manuscript. All authors contributed to editing the manuscript.

## Conflict of Interest

The authors declare that the research was conducted in the absence of any commercial or financial relationships that could be construed as a potential conflict of interest.

## References

[B1] Abramov I. Gordon J. Feldman O. Chavarga A. (2012). Sex and vision II: color appearance of monochromatic lights. *Biol. Sex Differ.* 3 1–16. 10.1186/2042-6410-3-21 22943488 PMC3483194

[B2] Aksoy S. Akçay H. G. Wassenaar T. (2010). Automatic mapping of linear woody vegetation features in agricultural landscapes using very high resolution imagery. *IEEE Trans. Geosci. Remote Sens.* 48 511–522. 10.1109/TGRS.2009.2027702

[B3] Barrett J. E. Virginia R. A. Wall D. H. Doran P. T. Fountain A. G. Welch K. A. (2008). Persistent effects of a discrete warming event on a polar desert ecosystem. *Glob. Chang. Biol.* 14 2249–2261. 10.1111/j.1365-2486.2008.01641.x

[B4] Bennett L. T. Judd T. S. Adams M. A. (2000). Close-range vertical photography for measuring cover changes in perennial grasslands. *J. Range Manag.* 53 634–641. 10.2458/azu_jrm_v53i6_bennett

[B5] Berberoglu S. Akin A. Atkinson P. M. Curran P. J. (2010). Utilizing image texture to detect land-cover change in Mediterranean coastal wetlands. *Int. J. Remote Sens.* 31 2793–2815. 10.1080/01431160903111077

[B6] Bergstrom D. M. (2017). Ecosystem shift after a hot event. *Nat. Ecol. Evol.* 1 1226–1227. 10.1038/s41559-017-0262-z 29046543

[B7] Blaschke T. (2004). “Object-based contextual image classification built on image segmentation,” in *Proceedings of the IEEE Workshop on Advances in Techniques for Analysis of Remotely Sensed Data, 2003*, (Greenbelt, MD: IEEE), 113–119. 10.1109/WARSD.2003.1295182

[B8] Blaschke T. (2010). Object based image analysis for remote sensing. *ISPRS J. Photogramm. Remote Sens.* 65 2–16. 10.1016/j.isprsjprs.2009.06.004 PMC3945831 24623958

[B9] Blaschke T. Strobl J. (2001). What’s wrong with pixels? Some recent developments interfacing remote sensing and GIS. *Geo Inform. Syst.* 14 12–17. 10.1364/AO.52.007629 24216667

[B10] Bollard-Breen B. Brooks J. D. Jones M. R. L. Robertson J. Betschart S. Kung O. (2015). Application of an unmanned aerial vehicle in spatial mapping of terrestrial biology and human disturbance in the McMurdo Dry Valleys, East Antarctica. *Polar Biol.* 38 573–578. 10.1007/s00300-014-1586-7

[B11] Booth D. T. Cox S. E. Johnson D. E. (2005b). Detection-threshold calibration and other factors influencing digital measurements of ground cover. *Rangel. Ecol. Manag.* 58 598–604. 10.2111/05-060R1.1

[B12] Booth D. T. Cox S. E. Fifield C. Phillips M. Williamson N. (2005a). Image analysis compared with other methods for measuring ground cover. *Arid. L. Res. Manag.* 19 91–100. 10.1080/15324980590916486

[B13] Brown R. O. MacLeod D. I. A. (1997). Color appearance depends on the variance of surround colors. *Curr. Biol.* 7 844–849. 10.1016/S0960-9822(06)00372-1 9382808

[B14] Bunting P. Lucas R. (2006). The delineation of tree crowns in Australian mixed species forests using hyperspectral compact airborne spectrographic imager (CASI) data. *Remote Sens. Environ.* 101 230–248. 10.1016/j.rse.2005.12.015

[B15] Chen Z. Chen W. Leblanc S. G. Henry G. H. R. (2010). Digital photograph analysis for measuring percent plant cover in the Arctic. *Arctic* 63 315–326. 10.14430/arctic1495

[B16] Conchedda G. Durieux L. Mayaux P. (2007). “Object-based monitoring of land cover changes in mangrove ecosystems of Senegal,” in *Proceedings of the 2007 International Workshop on the Analysis of Multi-Temporal Remote Sensing Images*, (Leuven: IEEE), 1–6. 10.1109/MULTITEMP.2007.4293039

[B17] Congalton R. G. Green K. (2009). *Assessing the Accuracy of Remotely Sensed Data: Principles and Practices.* Boca Raton, FL: CRC Press.

[B18] Cserhalmi D. Nagy J. Kristóf D. Neidert D. (2011). Changes in a wetland ecosystem: a vegetation reconstruction study based on historical panchromatic aerial photographs and succession patterns. *Folia Geobot.* 46 351–371. 10.1007/s12224-011-9099-4

[B19] Drǎguţ L. Tiede D. Levick S. R. (2010). ESP: a tool to estimate scale parameter for multiresolution image segmentation of remotely sensed data. *Int. J. Geogr. Inf. Sci.* 24 859–871. 10.1080/13658810903174803

[B20] Dunn J. L. Robinson S. A. (2006). Ultraviolet B screening potential is higher in two cosmopolitan moss species than in a co-occurring Antarctic endemic moss: implications of continuing ozone depletion. *Glob. Chang. Biol.* 12 2282–2296. 10.1111/j.1365-2486.2006.01283.x

[B21] Ehlers M. Gaehler M. Janowsky R. (2006). Automated techniques for environmental monitoring and change analyses for ultra high resolution remote sensing data. *Photogramm. Eng. Remote Sens.* 72 835–844. 10.14358/PERS.72.7.835

[B22] Elzinga C. Salzer D. Willoughby J. (1998). “Measuring & monitering plant populations,” in *U.S. Bureau of Land Management Papers*, 17 Available online at: http://digitalcommons.unl.edu/usblmpub/17 (accessed April 20, 2020).

[B23] Fensham R. J. Fairfax R. J. (2002). Aerial photography for assessing vegetation change: a review of applications and the relevance of findings for Australian vegetation history. *Aust. J. Bot.* 50 415–429. 10.1071/BT01032

[B24] Gorrod E. J. Keith D. A. (2009). Observer variation in field assessments of vegetation condition: implications for biodiversity conservation. *Ecol. Manag. Restor.* 10 31–40. 10.1111/j.1442-8903.2009.00437.x

[B25] Greenwood D. L. Weisberg P. J. (2009). GIS-based modeling of pinyon-juniper woodland structure in the great basin. *For. Sci.* 55 1–12. 10.17221/96/2008-jfs

[B26] Hájek F. (2008). Process-based approach to automated classification of forest structures using medium format digital aerial photos and ancillary GIS information. *Eur. J. For. Res.* 127 115–124. 10.1007/s10342-007-0188-0

[B27] Hall F. C. (2002). *Photo Point Monitoring Handbook: Part B-Concepts and Analysis. Portland, OR.* Available online at: http://npshistory.com/publications/interdisciplinary/im/pnw-gtr526b.pdf (accessed May 2, 2020).

[B28] Hay G. J. Castilla G. Wulder M. A. Ruiz J. R. (2005). An automated object-based approach for the multiscale image segmentation of forest scenes. *Int. J. Appl. Earth Obs. Geoinf.* 7 339–359. 10.1016/j.jag.2005.06.005

[B29] Haywood A. Stone C. (2011). Semi-automating the stand delineation process in mapping natural eucalypt forests. *Aust. For.* 74 13–22. 10.1080/00049158.2011.10676341

[B30] Howard-Williams C. Peterson D. Lyons W. B. Cattaneo-Vietti R. Gordon S. (2006). Measuring ecosystem response in a rapidly changing environment: the latitudinal gradient project. *Antarct. Sci.* 18 465–471. 10.1017/S0954102006000514

[B31] IPCC (2014). “Climate change 2014: impacts, adaptation, and vulnerability. Part A: global and sectoral aspects,” in *Contribution of Working Group II to the Fifth Assessment Report of the Intergovernmental Panel on Climate Change*, eds Field C. B. Barros V. R. Dokken D. J. Mach K. J. Mastrandrea M. D. Bilir T. E. (Cambridge: Cambridge University Press).

[B32] Jawak S. D. Raut D. A. Luis A. J. (2015). Iterative spectral index ratio exploration for object-based image analysis of Antarctic coastal oasis using high resolution satellite remote sensing data. *Aquat. Procedia* 4 157–164. 10.1016/j.aqpro.2015.02.022

[B33] Jensen J. R. (2013). *Remote Sensing of the Environment: An Earth Resource Perspective*, 2nd Edn Harlow: Pearson Education Limited.

[B34] Jobin B. Labrecque S. Grenier M. Falardeau G. (2008). Object-based classification as an alternative approach to the traditional pixel-based classification to identify potential habitat of the Grasshopper Sparrow. *Environ. Manage.* 41 20–31. 10.1007/s00267-007-9031-0 17985180

[B35] Johansson P. Thor G. (2008). Lichen species density and abundance over ten years in permanent plots in inland Dronning Maud Land, Antarctica. *Antarct. Sci.* 20 115–121. 10.1017/S0954102007000855

[B36] Kim M. Warner T. A. Madden M. Atkinson D. S. (2011). Multi-scale GEOBIA with very high spatial resolution digital aerial imagery: Scale, texture and image objects. *Int. J. Remote Sens.* 32 2825–2850. 10.1080/01431161003745608

[B37] Ko D. Bristow N. Greenwood D. Weisberg P. (2009). Canopy cover estimation in semiarid woodlands: comparison of field-based and remote sensing methods. *For. Sci.* 55 132–141. 10.1093/forestscience/55.2.132

[B38] Kolyaie S. Treier U. A. Watmough G. R. Madsen B. Bøcher P. K. Psomas A. (2019). Transferability and the effect of colour calibration during multi-image classification of Arctic vegetation change. *Polar Biol.* 42 1227–1239. 10.1007/s00300-019-02491-7

[B39] Laliberte A. S. Rango A. (2011). Image processing and classification procedures for analysis of sub-decimeter imagery acquired with an unmanned aircraft over arid rangelands. *GIScience Remote Sens.* 48 4–23. 10.2747/1548-1603.48.1.4

[B40] Laliberte A. S. Browningb D. M. Herrickb J. E. Gronemeyera P. (2010). Hierarchical object-based classification of ultra-high-resolution digital mapping camera (DMC) imagery for rangeland mapping and assessment. *J. Spat. Sci.* 55 101–115. 10.1080/14498596.2010.487853

[B41] Laliberte A. S. Fredrickson E. L. Rango A. (2007a). Combining decision trees with hierarchical object-oriented image analysis for mapping arid rangelands. *Photogramm. Eng. Remote Sensing* 73 197–207. 10.14358/PERS.73.2.197

[B42] Laliberte A. S. Rango A. Herrick J. E. Fredrickson E. L. Burkett L. (2007b). An object-based image analysis approach for determining fractional cover of senescent and green vegetation with digital plot photography. *J. Arid Environ.* 69 1–14. 10.1016/j.jaridenv.2006.08.016

[B43] Lathrop R. G. Montesano P. Haag S. (2006). A multi-scale segmentation approach to mapping seagrass habitats using airborne digital camera imagery. *Photogramm. Eng. Remote Sens.* 72 665–675. 10.14358/PERS.72.6.665

[B44] Lawley V. Lewis M. Clarke K. Ostendorf B. (2016). Site-based and remote sensing methods for monitoring indicators of vegetation condition: an Australian review. *Ecol. Indic.* 60 1273–1283. 10.1016/j.ecolind.2015.03.021

[B45] Lee J. R. Raymond B. Bracegirdle T. J. Chadès I. Fuller R. A. Shaw J. D. (2017). Climate change drives expansion of Antarctic ice-free habitat. *Nature* 547 49–54. 10.1038/nature22996 28658207

[B46] Liu D. Xia F. (2010). Assessing object-based classification: advantages and limitations. *Remote Sens. Lett.* 1 187–194. 10.1080/01431161003743173

[B47] Lovelock C. E. Robinson S. A. (2002). Surface reflectance properties of antarctic moss and their relationship to plant species, pigment composition and photosynthetic function. *Plant Cell Environ.* 25 1239–1250. 10.1046/j.1365-3040.2002.00916.x

[B48] Luscier J. D. Thompson W. L. Wilson J. M. Gorham B. E. Dragut L. D. (2006). Using digital photographs and object−based image analysis to estimate percent ground cover in vegetation plots. *Front. Ecol. Environ.* 4:408 10.1890/1540-929520064[408:UDPAOI]2.0.CO;2

[B49] Malenovský Z. Lucieer A. King D. H. Turnbull J. D. Robinson S. A. (2017). Unmanned aircraft system advances health mapping of fragile polar vegetation. *Methods Ecol. Evol.* 8 1842–1857. 10.1111/2041-210X.12833

[B50] Malenovský Z. Turnbull J. D. Lucieer A. Robinson S. A. (2015). Antarctic moss stress assessment based on chlorophyll content and leaf density retrieved from imaging spectroscopy data. *New Phytol.* 208 608–624. 10.1111/nph.13524 26083501

[B51] McCarthy D. P. Zaniewski K. (2001). Digital analysis of lichen cover: a technique for use in lichenometry and licnenology. *Arctic Antarct. Alp. Res.* 33 107–113. 10.1080/15230430.2001.12003411

[B52] Michaels A. Power A. G. (2011). *Long-Term Ecological Research Program. A Report of the 30 Year Review Committee.* Available online at: papers3://publication/uuid/1898E571-88FC-4FC1-A3A7-48AAC2430420 (accessed April 20, 2020).

[B53] Michel P. Mathieu R. Mark A. F. (2010). Spatial analysis of oblique photo-point images for quantifying spatio-temporal changes in plant communities. *Appl. Veg. Sci.* 13 173–182. 10.1111/j.1654-109X.2009.01059.x

[B54] Pecl G. T. Araújo M. B. Bell J. D. Blanchard J. Bonebrake T. C. Chen I.-C. (2017). Biodiversity redistribution under climate change: Impacts on ecosystems and human well-being. *Science* 355:eaai9214. 10.1126/science.aai9214 28360268

[B55] Post A. (1990). Photoprotective pigment as an adaptive strategy in the antarctic moss Ceratodon purpureus. *Polar Biol.* 10 241–245. 10.1007/BF00238420

[B56] Pringle R. M. Syfert M. Webb J. K. Shine R. (2009). Quantifying historical changes in habitat availability for endangered species: Use of pixel- and object-based remote sensing. *J. Appl. Ecol.* 46 544–553. 10.1111/j.1365-2664.2009.01637.x

[B57] Robinson S. A. King D. H. Bramley-Alves J. Waterman M. J. Ashcroft M. B. Wasley J. (2018). Rapid change in East Antarctic terrestrial vegetation in response to regional drying. *Nat. Clim. Chang.* 8 879–884. 10.1038/s41558-018-0280-0

[B58] Robinson S. A. Klekociuk A. R. King D. H. Pizarro Rojas M. Zúñiga G. E. Bergstrom D. M. (2020). The 2019/2020 summer of Antarctic heatwaves. *Glob. Chang. Biol.* 10.1111/gcb.15083 [Epub ahead of print]. 32227664

[B59] Rogers G. F. Turner R. M. Malde H. E. (1983). “Using matched photographs to monitor resource change,” in *Proceedings, International Conference Renewable Resource Inventories for Monitoring Changes and Trend*, eds Bell J. F. Atterbury T. (Corvallis, OR: College of Forestry), 90–92.

[B60] Ryan-Colton E. (2007). *Long-Term Monitoring of the Impacts of Climate Change on Antarctic Terrestrial Communities: Baseline and Method Developments.* Wollongong NSW: University of Wollongong.

[B61] Stow D. A. Hope A. McGuire D. Verbyla D. Gamon J. Huemmrich F. (2004). Remote sensing of vegetation and land-cover change in Arctic Tundra Ecosystems. *Remote Sens. Environ.* 89 281–308. 10.1016/j.rse.2003.10.018

[B62] Torres-Sánchez J. López-Granados F. Peña J. M. (2015). An automatic object-based method for optimal thresholding in UAV images: application for vegetation detection in herbaceous crops. *Comput. Electron. Agric.* 114 43–52. 10.1016/j.compag.2015.03.019

[B63] Trussell H. J. Vrhel M. J. (2008). *Fundamentals of Digital Imaging.* Cambridge: Cambridge University Press.

[B64] Turner D. Lucieer A. Watson C. (2012). An automated technique for generating georectified mosaics from ultra-high resolution unmanned aerial vehicle (UAV) imagery, based on structure from motion (SfM) point clouds. *Remote Sens.* 4 1392–1410. 10.3390/rs4051392

[B65] Turner D. Lucieer A. Malenovský Z. King D. H. Robinson S. A. (2014). Spatial co-registration of ultra-high resolution visible, multispectral and thermal images acquired with a micro-UAV over Antarctic Moss Beds. *Remote Sens.* 6 4003–4024. 10.3390/rs6054003

[B66] Turner D. Lucieer A. Malenovský Z. King D. H. Robinson S. A. (2018). Assessment of Antarctic moss health from multi-sensor UAS imagery with random forest modelling. *Int. J. Appl. Earth Obs. Geoinf.* 68 168–179. 10.1016/j.jag.2018.01.004

[B67] Van Coillie F. M. B. Gardin S. Anseel F. Duyck W. Verbeke L. P. C. De Wulf R. R. (2014). Variability of operator performance in remote-sensing image interpretation: the importance of human and external factors. *Int. J. Remote Sens.* 35 754–778. 10.1080/01431161.2013.873152

[B68] Verbyla D. L. (2002). *Practical GIS Analysis.* Milton Park: Taylor & Francis.

[B69] Wasley J. Robinson S. A. Lovelock C. E. Popp M. (2006). Some like it wet – biological characteristics underpinning tolerance of extreme water stress events in Antarctic bryophytes. *Funct. Plant Biol.* 33 443–455. 10.1071/FP05306 32689251

[B70] Wasley J. Robinson S. A. Turnbull J. D. King D. H. Wanek W. Popp M. (2012). Bryophyte species composition over moisture gradients in the Windmill Islands, East Antarctica: development of a baseline for monitoring climate change impacts. *Biodiversity* 13 257–264. 10.1080/14888386.2012.712636

[B71] Waterman M. J. Bramley-Alves J. Miller R. E. Keller P. A. Robinson S. A. (2018). Photoprotection enhanced by red cell wall pigments in three East Antarctic mosses. *Biol. Res.* 51 13–49. 10.1186/s40659-018-0196-1 30463628 PMC6247747

[B72] Whiteside T. G. Boggs G. S. Maier S. W. (2011). Comparing object-based and pixel-based classifications for mapping savannas. *Int. J. Appl. Earth Obs. Geoinf.* 13 884–893. 10.1016/j.jag.2011.06.008

[B73] Yu Q. Yu Q. Gong P. Clinton N. Biging G. Kelly M. (2006). Object-based detailed vegetation classification with airborne high spatial resolution remote sensing imagery. *Photogramm. Eng. Remote Sens.* 72 799–811.

[B74] Zhang L. Li L. J. Liang L. Q. Li J. Y. (2007). Monitoring of vegetation coverage based on high-resolution images. *For. Stud. China* 9 256–261. 10.1007/s11632-007-0040-0

