# Supplementary figures and images for: Semi-Automated Analysis of Digital Photographs for Monitoring East Antarctic Vegetation
Source: Front Plant Sci. 2020 Jun 9;11:766. doi: 10.3389/fpls.2020.00766 (PMC7296125; doi:10.3389/fpls.2020.00766)

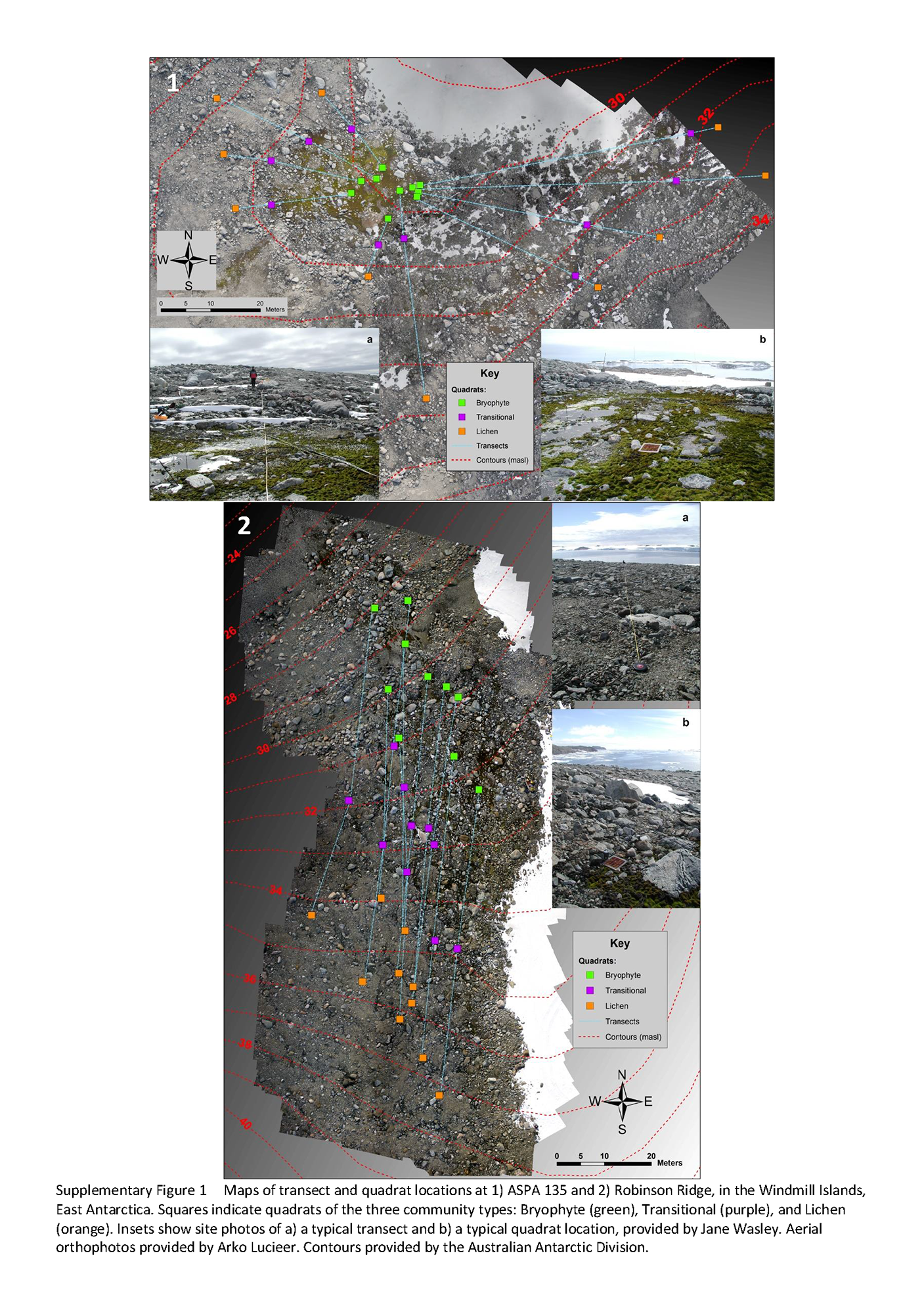

Supplement: Supplementary file 1 [file Image_1.TIF]

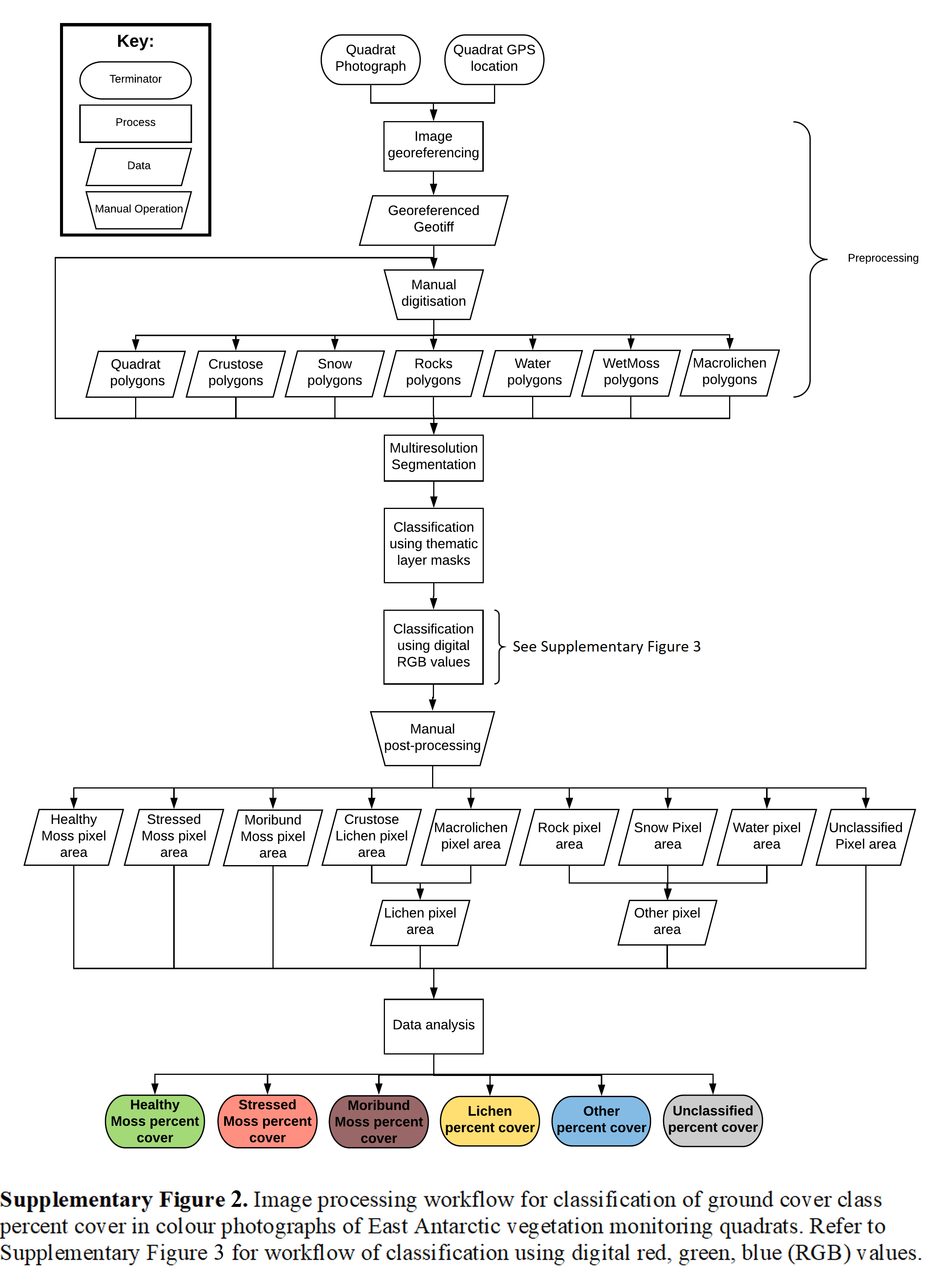

Supplement: Supplementary file 2 [file Image_2.tif]

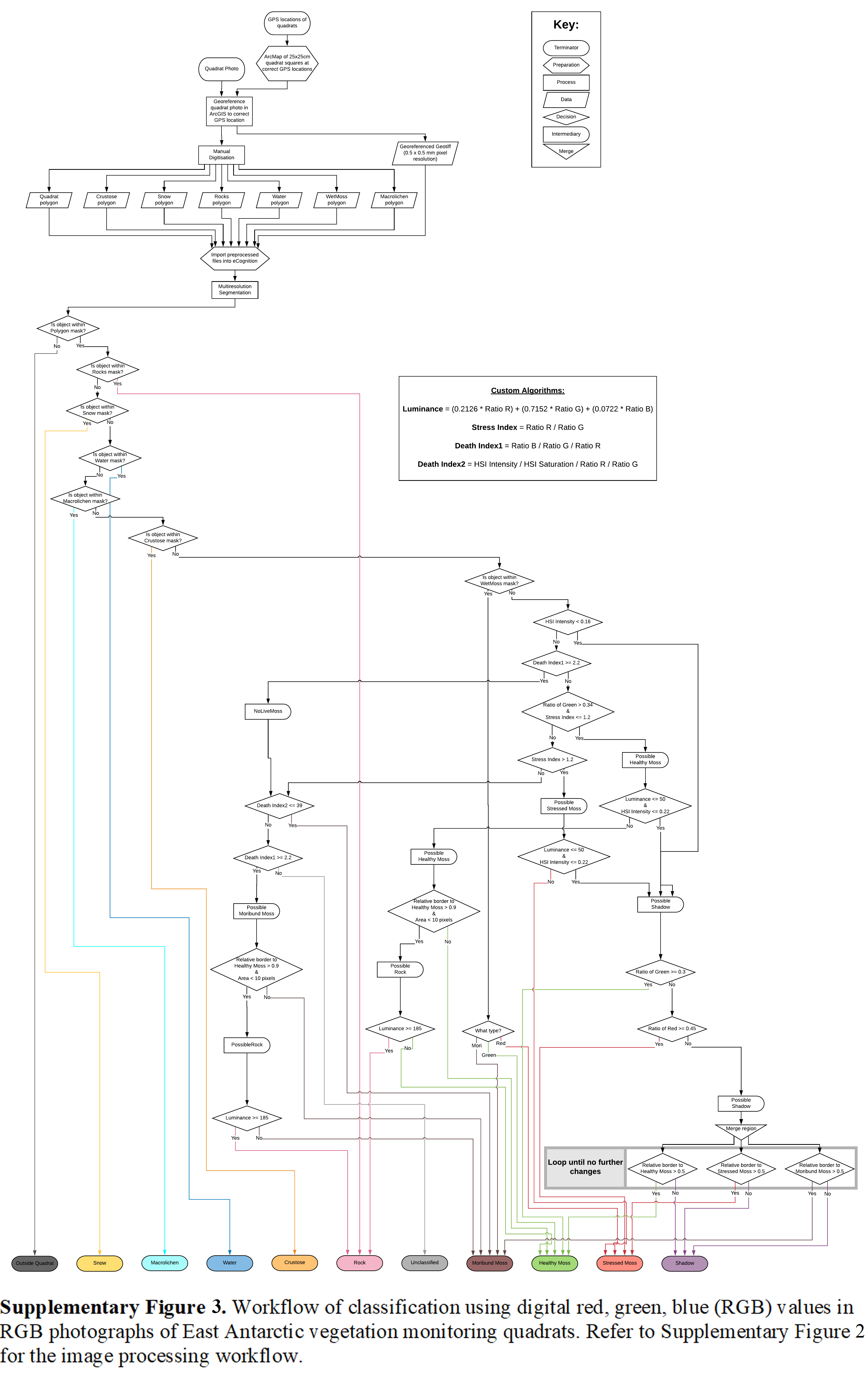

Supplement: Supplementary file 3 [file Image_3.TIF]
