# Supplementary material for: Semi-Automated Analysis of Digital Photographs for Monitoring East Antarctic Vegetation
Source: Front Plant Sci. 2020 Jun 9;11:766. doi: 10.3389/fpls.2020.00766 (PMC7296125; doi:10.3389/fpls.2020.00766)
Supplement: Supplementary file 4 [file Table_1.DOCX]

Supplementary Material

Supplementary Table 1 Field dates and digital camera specifications for each field season, for Windmill Islands fieldwork from 2003 to 2014.

| **Season** | **Field dates** | **Camera** | **Image Resolution (pixels)** |
| --- | --- | --- | --- |
| 2003 | Robinson Ridge: 24/01/2003  ASPA 135: 11/02/2003 | Olympus C3000Z | 2048 x 1536 |
| 2008 | Robinson Ridge: 14/01/2008  ASPA 135: 15/01/2008 | Olympus µ795SW | 3072 x 2304 |
| 2011 | Robinson Ridge: 24/02/2011  ASPA 135: 21 & 26/02/2011 | Sony DSC-HX5V | 3648 x 2736 |
| 2012 | Robinson Ridge: 14/01/2012  ASPA 135: 20/01/2012 | Sony DSC-HX9V | 4608 x 3456 |
| 2013 | Robinson Ridge: 16 & 17/01/2013  ASPA 135: 18 & 27/01/2013 & 04/02/2013 | Sony DSC-HX9V | 4608 x 3456 |
| 2014 | Robinson Ridge: 20/01/2014  ASPA 135: 15/01/2014 | Sony NEX-7 | 6000 x 4000 |
